# Palmitic Acid Methyl Ester and Its Relation to Control of Tone of Human Visceral Arteries and Rat Aortas by Perivascular Adipose Tissue

**DOI:** 10.3389/fphys.2018.00583

**Published:** 2018-05-23

**Authors:** Ning Wang, Artur Kuczmanski, Galyna Dubrovska, Maik Gollasch

**Affiliations:** ^1^Experimental and Clinical Research Center, Charité – Universitätsmedizin Berlin and Max-Delbrück Center for Molecular Medicine in the Helmholtz Association (MDC), Berlin, Germany; ^2^HELIOS Klinikum Berlin-Buch, Berlin, Germany; ^3^Medical Clinic of Nephrology and Internal Intensive Care, Charité – Universitätsmedizin Berlin, Berlin, Germany

**Keywords:** XE991, adipocyte-derived relaxing factor (ADRF), perivascular adipose tissue (PVAT), KCNQ channels, K_v_ channels

## Abstract

**Background:** Perivascular adipose tissue (PVAT) exerts anti-contractile effects on visceral arteries by release of various perivascular relaxing factors (PVRFs) and opening voltage-gated K^+^ (K_v_) channels in vascular smooth muscle cells (VSMCs). Palmitic acid methyl ester (PAME) has been proposed as transferable PVRF in rat aorta. Here, we studied PVAT regulation of arterial tone of human mesenteric arteries and clarified the contribution of K_v_ channels and PAME in the effects.

**Methods:** Wire myography was used to measure vasocontractions of mesenteric artery rings from patients undergoing abdominal surgery. Isolated aortic rings from Sprague-Dawley rats were studied for comparison. PVAT was either left intact or removed from the arterial rings. Vasocontractions were induced by external high K^+^ (60 mM), serotonin (5-HT) or phenylephrine. PAME (10 nM−3 μM) was used as vasodilator. K_v_ channels were blocked by XE991, a K_v_7 (KCNQ) channel inhibitor, or by 4-aminopyridine, a non-specific K_v_ channel inhibitor. PAME was measured in bathing solutions incubated with rat peri-aortic or human visceral adipose tissue.

**Results:** We found that PVAT displayed anti-contractile effects in both human mesenteric arteries and rat aortas. The anti-contractile effects were inhibited by XE991 (30 μM). PAME (EC_50_ ~1.4 μM) was capable to produce relaxations of PVAT-removed rat aortas. These effects were abolished by XE991 (30 μM), but not 4-aminopyridine (2 mM) or NDGA (10 μM), a lipoxygenases inhibitor. The cytochrome P450 epoxygenase inhibitor 17-octadecynoic acid (ODYA 10 μM) and the soluble epoxide hydrolase inhibitor 12-(3-adamantan-1-ylureido)-dodecanoic acid (AUDA 10 μM) slightly decreased PAME relaxations. PAME up to 10 μM failed to induce relaxations of PVAT-removed human mesenteric arteries. 5-HT induced endogenous PAME release from rat peri-aortic adipose tissue, but not from human visceral adipose tissue.

**Conclusions:** Our data also suggest that K_v_7 channels are involved in the anti-contractile effects of PVAT on arterial tone in both rat aorta and human mesenteric arteries. PAME could contribute to PVAT relaxations by activating K_v_7 channels in rat aorta, but not in human mesenteric arteries.

## Introduction

Perivascular adipose tissue (PVAT), which surrounds the aorta, its vascular branches and many other arteries, is now recognized as dynamic paracrine organ and important metabolic sensor (Szasz et al., 2013; Gil-Ortega et al., 2015; Gollasch, 2017). PVAT does not only provide mechanical protection to vessels but also regulates vascular function by releasing perivascular adipose relaxing factors (PVRFs), particularly a transferable adipocyte-derived relaxing factor (ADRF), which diminishes the contractile actions of vasoconstrictors such as phenylephrine (PE), serotonin (5-HT), angiotensin II and U46619 (Löhn et al., 2002; Yiannikouris et al., 2010). The anti-contractile effect of PVAT has been observed in both large and small arteries of rats, mice, pigs and humans (Bunker and Laughlin, 1985; Szasz and Webb, 2012; Gollasch, 2017). The anti-contractile effects of PVAT rely on the opening of K^+^ channels in vascular smooth muscle cells (VSMCs) (Tano et al., 2014). This action occurs without involvement of NO, prostaglandin I_2_ (prostacyclin) or endothelium-derived hyperpolarizing factor (EDHF) (Löhn et al., 2002; Li et al., 2013).

VSMC K_v_7 channels are considered to play a key role for vasodilation by ADRF released from PVAT (Gollasch, 2017). Consistently, the anti-contractile effects of PVAT are abolished by the K_v_7 channel blocker XE991 in rat and mouse visceral arteries (Löhn et al., 2002; Schleifenbaum et al., 2010; Tsvetkov et al., 2016b). Although, the exact nature of ADRF is unknown, adiponectin, Ang 1–7, H_2_S and palmitic acid methyl ester (PAME) have been proposed as ADRF candidates (Fang et al., 2009; Lee et al., 2011; Gu and Xu, 2013; Lynch et al., 2013). The effects of adiponectin on vascular tone are mediated by activation of calcium-activated K^+^ (BK_Ca_) channels on VSMCs and adipocytes and by endothelial mechanisms (Lynch et al., 2013; Baylie et al., 2017), or K_v_ channel-dependent mechanisms (Fésüs et al., 2007). PAME is one of the most abundant fatty acids in mammalian cells (Lau et al., 2017), and represents an endogenous naturally occurring fatty acid methyl ester (Fukuda et al., 1967). This compound has been reported to have the ability to inhibit Kupffer cells which are resident macrophages in the liver regulating inflammatory processes by secretion of TNF-alpha and NO (Cai et al., 2005). PAME is also known to exhibit anti-fibrotic effects (Fukunishi et al., 2011) and to act as potent vasodilator released in retina and myometrium (Lee et al., 2010, 2011; Crankshaw et al., 2014). A recent report identified PAME as novel, potent vasodilator released from PVAT in rat aorta, which exhibits vascular relaxation by opening K_v_ channels in smooth muscle cells (Lee et al., 2011). Although these findings suggest that PAME could represent a potential mediator in control of vasotonus and blood pressure in rats, the role of K_v_7 channels in PVAT regulation of human arterial tone and vasodilatory PAME effects remains to be established. Therefore, we tested the hypothesis that XE991-sensitive K_v_ (K_v_7) channels are involved in the anti-contractile effects of PVAT on human mesenteric arteries. Furthermore, we investigated the contribution of endogenous PAME to PVAT regulation of arterial tone in human mesenteric arteries and the role of K_v_7 channels in vasodilatory PAME effects. Isolated aortic rings from Sprague-Dawley rats were studied for comparison. Finally, we tested whether PAME might contribute to PVAT regulation of arterial tone by involving metabolism of endogenous lipid epoxides.

## Materials and methods

### Isometric contractions of rat vessels

The local animal review board of Berlin (LAGESO) approved all studies, according to American Physiological Society criteria. Male Sprague-Dawley rats (200–300 g, 8–10 weeks; Charles River, Sulzfeld/Berlin Germany) were killed, and the thoracic aortas were removed, and quickly transferred to cold (4°C) oxygenated (95% O_2_/5% CO_2_) physiological salt solution (PSS), and dissected into 2 mm rings, respectively. Perivascular fat and connective tissue were either removed [(–) fat] or left [(+) fat] intact as previously described. The rings were placed under force of 20 mN. The bath solution volume was 20 mL of a vessel myograph (Schuler tissue bath system, Hugo Sachs Elektronik, Freiburg, Germany). After 1 h equilibration, contractile force was measured isometrically using standard bath procedures and solutions as described (Dubrovska et al., 2004; Kohn et al., 2012; Brennan et al., 2016).

Cumulative concentration response curves were obtained for PAME (Löhn et al., 2002) in the presence and absence of the K^+^ channel or enzyme inhibitors, 10,10-bis(4-pyridinylmethyl)-9(10H)-anthracenone dihydrochloride (XE991); 4-aminopyridine (4-AP); nordihydroguaiaretic acid (NDGA); 17-octadecadiynoic acid (ODYA); or 12-(3-adamantan-1-ylureido)-dodecanoic acid (AUDA). Tension was expressed as a percentage of the steady-state tension (100%) obtained with isotonic external 60 mM KCl. To test for the presence of functional endothelium, rings were contracted with 1 μM PE and once the vessels reached a stable maximum tension, the vessels were stimulated with 10 μM acetylcholine (ACh) and relaxation was confirmed (>80%) (Löhn et al., 2002). In some rings, the endothelium was removed by gently abrading the luminal surface of the vessel with a stainless steel pin to determine the contribution of the endothelium to PAME relaxation. Functional endothelium was considered absent if 10 μM ACh did not produce relaxation (Löhn et al., 2002).

In bioassay experiments, we transferred aliquots of bath solution from aorta with PVAT incubated in a donor bath chamber to vessel preparations without PVAT in an acceptor bath chamber of the Schuler tissue bath system (Hugo Sachs Elektronik, Freiburg, Germany). Cumulative response curves were obtained in the presence and absence of 5-HT (total incubation time, 5 min). The volume of the solutions in the bath was 20 mL. In most experiments, transfer interval of aliquots was 15–20 min; the volume of the aliquots was 3 or 5 mL. Transfer of bath solution aliquots from aortic vessels without PVAT or fresh PSS did not affect contraction of vessel preparations without PVAT in the acceptor bath chamber (Löhn et al., 2002).

### Isometric contractions of human vessels

Procedures were performed in accordance with the ethics guidelines of the National Health and Medical Research Council of Germany. All patients provided informed consent for participation in this study. Mesenteric tissue was taken from 12 patients (1 female, 11 males) undergoing surgical treatment of bowel carcinoma or inflammatory bowel disorders [colon cancer (*n* = 3), sigma cancer (*n* = 4), rectal cancer (*n* = 1), colon adenoma (*n* = 1), Crohn's disease (*n* = 1), and sigmoid diverticulitis (*n* = 1)]. The mean age of the patients was 69 years (range: 46–80), the mean BMI of the patients was 25 kg/m^2^ (range: 20–30 kg/m^2^), which is expected for the general population, since adopting the WHO classification is that ~50% or more of the general adult population will always be in the overweight range (now pre-obese, BMI 25–30 kg/m^2^), at least in the US and Western Europe (Nuttall, 2015). Few patients were taking drugs, including β-blockers (*n* = 4), angiotensin-converting-enzyme inhibitors (*n* = 2), metformin (*n* = 2), calcium channel blocker (*n* = 1), diuretic (*n* = 1), or fibrates (*n* = 1). Immediately after lower intestinal surgery, mesenteric arteries were excised from resected mesenteric tissue, and quickly transferred to cold (4°C) oxygenated (95% O_2_/5% CO_2_) PSS, and dissected into 1 mm rings. PVAT was either removed [(−) fat] or left [(+) fat] intact as previously described (Schleifenbaum et al., 2014). Each ring was positioned between two stainless steel wires in a 5-mL organ bath of a Small Vessel Myograph (DMT 610M; Danish Myo Technology, Denmark) (Tsvetkov et al., 2016a). The organ bath was filled with PSS. The bath solution was continuously oxygenated with a gas mixture of 95% O_2_ and 5% CO_2_, and kept at 37°C (pH 7.4). The rings were placed under force of 3 mN. The software Chart5 (AD Instruments Ltd. Spechbach, Germany) was used for data acquisition and display. The rings were pre-contracted with 60 mM KCl and equilibrated until a stable resting tension was acquired. Chemicals were added to the bath solution if not indicated otherwise. Vessels were pre-contracted with either 5-HT or phenylephrine. All chemicals were added to the bath solution (PSS).

### Gas chromatography/mass spectrometry (GC/MS) analysis

PAME measurements were performed by Shanghai Ingeer Certification Assessment Co, Ltd (ICAS, Shanghai, China). GC/MS analysis was performed using an Agilent ChemStation. For determination of endogenous PAME concentrations in bath solutions, rat peri-aortic and human visceral adipose tissue (3 g each) were incubated in 15 mL-Eppendorf tubes with 10 mL PSS solutions, with or without 5-HT 5 μM (30 min, in 37°C water bath). PSS was oxygenated (95% O_2_/5% CO_2_) for 30 min before use. After removal of adipose tissue, the PSS solution were dissolved in hexane (1:3 volume ratio), extracted and vortexed. Next, 1 mL water was added to the solution. In order to ensure that the concentration of PAME between the aqueous and the lipophilic phase was in equilibrium the samples were shaken by hand for 4 min. Thereafter, the phases were separated by centrifugation and the lipophilic hexane phase containing fatty acid methyl esters was removed and dried under nitrogen. The fatty acid methyl ester residues were re-dissolved in 50 μL hexane and transferred into an autosampler vial. Samples were analyzed by using a fully automated Agilent 7890A-5977B system equipped with a flame ionization detector. Peaks of re-dissolved PAME were identified by comparison with PAME standard and their nominal concentrations were determined (Yi et al., 2014; Siegert et al., 2017). (+) Fat masses were measured in rat aortic (2 mm) and human mesenteric artery (1 mm) rings (*n* = 6 each) to calculate magnitudes of effective [PAME] in the 20 or 5 mL myograph bath chambers, respectively.

### Materials and statistics

The composition of PSS (in mM) was 119 NaCl, 4.7 KCl, 1.2 KH_2_PO4, 25 NaHCO_3_, 1.2 MgSO_4_, 11.1 glucose, and 1.6 CaCl_2_. The composition of 60 mM KCl solution (in mM) was 59 NaCl, 60 KCl, 1.2 KH_2_PO4, 25 NaHCO_3_, 1.2 MgSO_4_, 11.1 glucose, and 1.6 CaCl_2._ All salts were purchased from Sigma Aldrich (Schnelldorf, Germany). XE991 was purchased from Tocris (Bristol, UK). 5-HT, phenylephrine (PE), 4-aminopyridine (4-AP), nordihydroguaiaretic acid (NDGA), 17-octadecynoic acid (ODYA), 12-(3-adamantan-1-ylureido)-dodecanoic acid (AUDA) were purchased from Sigma Aldrich (Schnelldorf, Germany). PAME were purchased from Cayman Chemical (Ann Arbor, Michigan, USA).

**Figure 1:**
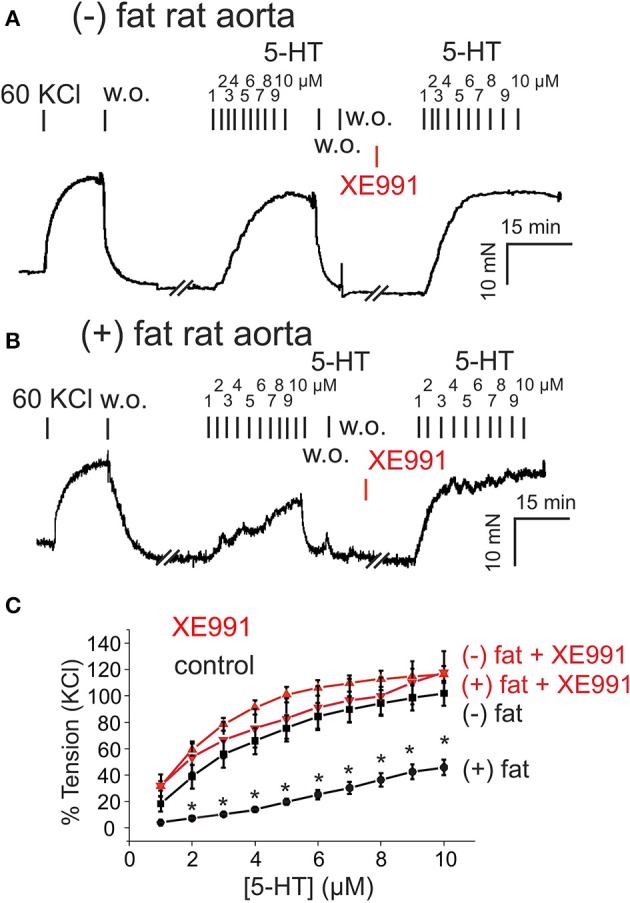
Presence of anti-contractile effects of perivascular adipose tissue (PVAT) in rat aorta and their inhibition by XE991. **(A,B)** Original recordings showing inhibition of the anti-contractile effects of PVAT by XE991. Incubation of (–) fat aortic rings with 30 μM XE-991 **(A)** had no effect on the contractile response. Incubation of (+) fat aortic rings with 30 μM XE991 **(B)** increased the contractile response to 5-HT. **(C)** Concentration-response curves for 5-HT (1–10 μM) in (+) fat vessels in the presence (*n* = 6) and absence of XE991 (*n* = 6) and in (–) fat vessels in the presence (*n* = 12) and absence of XE991 (*n* = 6). ^*^p < 0.05 for (+) fat control vessels compared to all other groups each, 60 KCl, 60 mM KCl.

**Figure 2:**
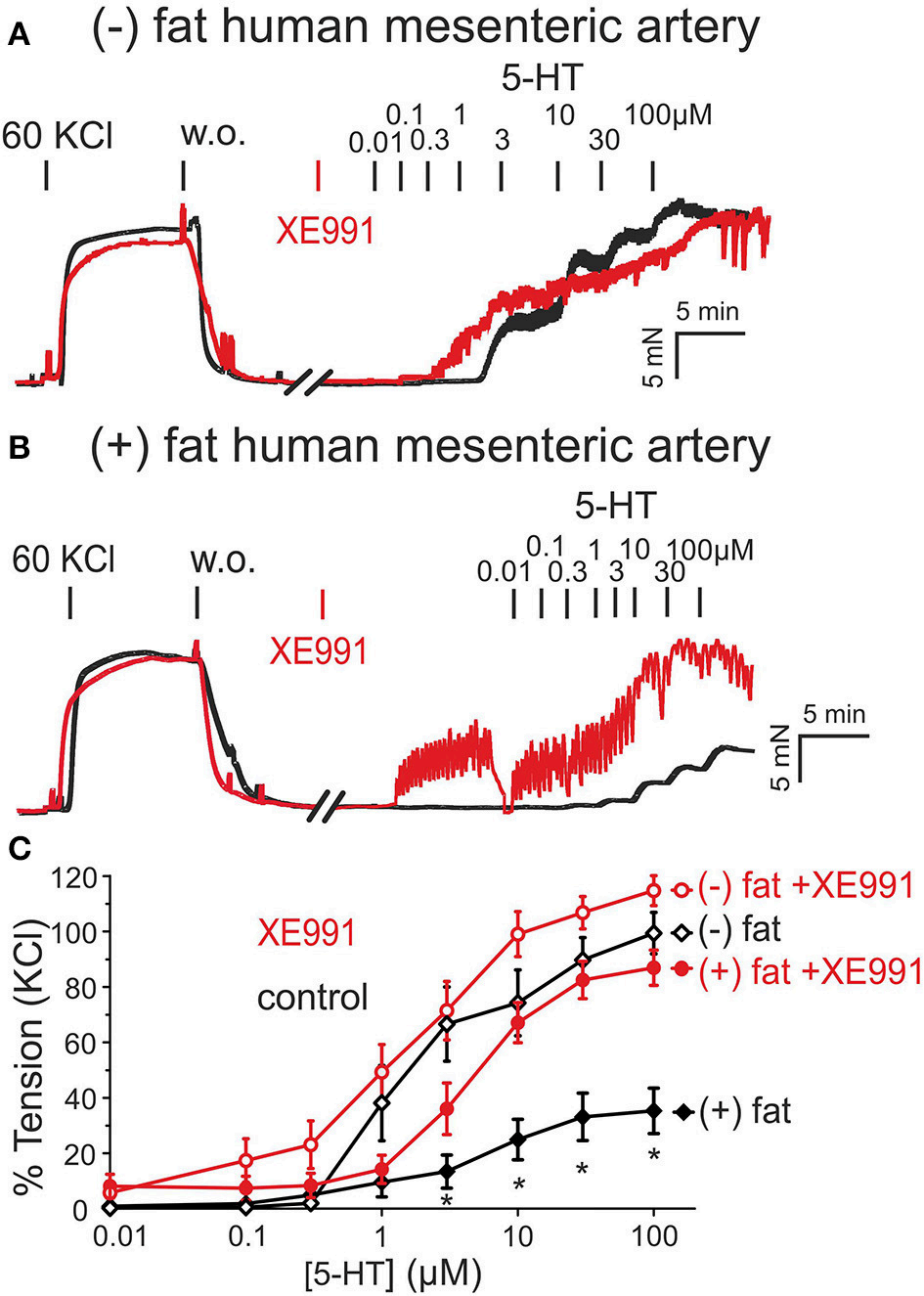
Presence of anti-contractile effects of PVAT in human mesenteric arteries and their inhibition by XE991. **(A,B)** Original recordings showing inhibition of the anti-contractile effects of PVAT by XE991 (30 μM). Recordings are shown in the presence (red curves) and absence (black curves) of XE991. XE991 had no effects on 5-HT contractions in (–) fat rings **(A)**, but increased 5-HT contractions in (+) fat rings **(B)**. **(C)** Concentration-response curves for 5-HT (0.01–100 μM) in (+) fat vessels in the presence (*n* = 13) and absence of XE991 (*n* = 15) and in (–) fat vessels in the presence (*n* = 10) and absence of XE991 (*n* = 10). ^*^p < 0.05, *p* < 0.05 for (+) fat control vessels compared to all other groups each.

Data were analyzed by Prism version 5.0 (GraphPad Software, La Jolla, California, USA) and were shown as mean ± SD or mean ± SEM. Paired, unpaired Student's *t*-tests or one-way ANOVA were used as appropriate. In Figures 1C, 2C, statistical significance was determined by two-way ANOVA or repeated-measures two-way ANOVA, followed by Bonferroni *post-hoc* test, and using Prism 6 software. A value of *P* < 0.05 was considered statistically significant; n represents the number of arteries tested.

## Results

### Contraction of rat aortas and human mesenteric arteries with and without PVAT under K_v_7 channel inhibition

We first investigated the role of K_v_7 channels in the anti-contractile effects of PVAT in rat aortas. Rat aortic rings without PVAT [(–) fat] showed stronger contractions to 5-HT (relative increase, 60–100% between 2 and 10 μM 5-HT) than vessels with PVAT [(+) fat] (Figure 1). Incubation of the vessels with the K_v_7 channel inhibitor XE991 (30 μM, 30 min) inhibited the anti-contractile effects of PVAT mediated by transferable ADRF in rat aortic rings (Figures 1B,C, Supplementary Figures 1). XE991 slightly (<20%) increased contractions of (–) fat rat aortic rings in response to 5-HT, but there was no difference between contractions of (–) fat and (+) fat rings in response to 5-HT (Figures 1A,C). Contraction of (–) fat and (+) fat aortic rings obtained by 60 mM KCl containing PSS were not different (13.07 ± 1.29 mN, *n* = 16 vs. 10.81 ± 1.19 mN, *n* = 14, *P* > 0.05, respectively).

Similar results were observed in human visceral arteries. Human mesenteric arteries without PVAT [(−) fat] showed significantly stronger contractions in response to 5-HT than vessels with PVAT [(+) fat] (Figure 2). Incubation of the vessel rings with XE991 (30 μM, 30 min) inhibited the anti-contractile effects of PVAT (Figures 2B,C). XE991 did not affect 5-HT-induced contractions in (–) fat human mesenteric artery rings (Figures 2A,C).

Similar data were obtained when vessels were contracted with phenylephrine (Supplementary Figures 2). These data suggest that PVAT displays anti-contractile effects in both rat aortas and human mesenteric arteries mediated by XE991-sensitive K_v_7 channels, and occur independently of the vasoconstrictor agonists used, i.e., serotoninergic or alpha-adrenergic agonists. Contraction of (–) fat and (+) fat human mesenteric arterial rings obtained by 60 mM KCl containing PSS were not different (19.46 ± 3.60 mN, *n* = 10 vs. 21.00 ± 2.15 mN, *n* = 12, *P* > 0.05, respectively).

### Pame relaxations and effects of NDGA, ODYA, and AUDA

**Figure 3:**
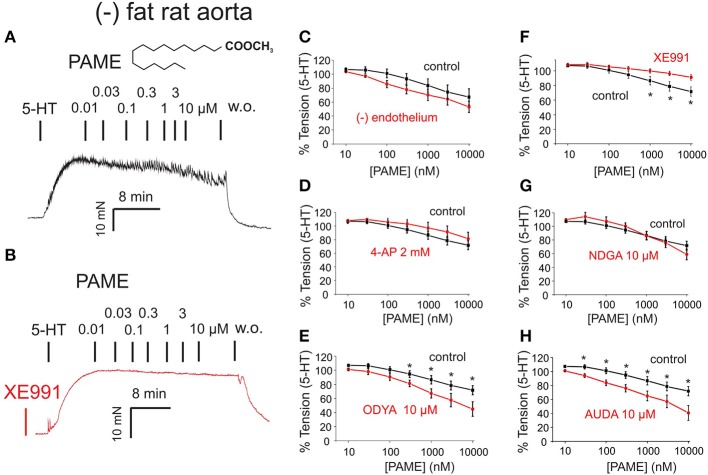
Effects of palmitic acid methyl ester (PAME, 0.01–10 μM) on rat aorta. The effects are shown in the absence (control, black curves) and presence (red curves) of inhibitors of K_v_ channels [4-aminopiridine (4-AP) 2 mM] or XE991 30 μM, lipoxygenase (LOX 15,12,5) (nordihydroguaiaretic acid (NDGA) 10 μM), cytochrome P450 (CYP) [17-octadecynoic acid (17-ODYA) 10 μM], and soluble epoxide hydrolase (sEH) [12-(3-adamantan-1-yl-ureido)dodecanoic acid (AUDA) 10 μM]. The effects were studied in the presence (+) and absence (–) of endothelium in 2 μM 5-HT precontracted (–) fat aortic rings. **(A,B)** Original recordings demonstrating the relaxant effect of PAME (0.01–10 μM) and inhibition of this effect **(B)** by pre-treatment of the vessels with 30 μM XE991. **(C)** Concentration-response curves for PAME in (–) fat aortic rings in the presence (Control) (*n* = 8) and absence (–) of the endothelium (*n* = 6). **(D)** Concentration-response curves for PAME in the absence (*n* = 13) and presence (*n* = 7) of 2 mM 4-AP in (–) fat aortic rings. **(E)** Concentration-response curves for PAME in non-treated (*n* = 13) and 10 μM 17-ODYA treated (*n* = 6) (–) fat aortic rings. ^*^p < 0.05. **(F)** Concentration-response curves for PAME in the absence (*n* = 6) and presence (*n* = 6) of 30 μM XE991 in (–) fat aortic rings. ^*^p < 0.05. **(G)** Concentration-response curves for PAME in non-treated (*n* = 13) and 10 μM NDGA (*n* = 6) treated (–) fat aortic rings. **(H)** Concentration-response curves for PAME in non-treated (*n* = 13) and 10 μM AUDA treated (*n* = 6) (–) fat aortic rings. Data are expressed as mean ± SEM. ^*^p < 0.05.

**Figure 4:**
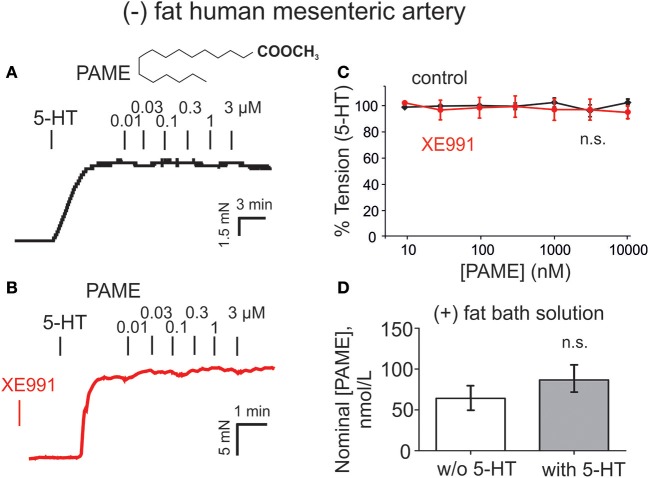
Lack of palmitic acid methyl ester (PAME, 0.01–10 μM) to induce relaxation in human mesenteric artery rings and measurements of PAME in bath solutions from human (+) fat mesenteric artery rings. **(A,B)** Original recordings demonstrating lack of vasorelaxant effects of PAME (0.01–3 μM) in (–) fat rings. Effects of PAME are shown in the absence (control, black curves) and presence (red curves) of XE991. The effects were studied on 2 μM 5-HT pre-contracted (–) fat mesenteric artery rings. **(C)** Concentration-response curves for PAME in (–) fat rings in the presence (Control) (PAME 0.01–3 μM, *n* = 12; PAME 10 μM, *n* = 8) and absence (PAME 0.01–3 μM, *n* = 12; PAME 10 μM, *n* = 8) of XE991. **(D)** Production of PAME shows no difference between bath solutions from human visceral adipose tissue incubated in the presence (*n* = 8) and absence of 5-HT (*n* = 8). w/o, without; n.s., not significant.

Exogenous PAME (EC_50_ ~1.4 μM; maximal relaxation E_max_ ~25%) was capable of producing relaxations of (–) fat rat aortas (Figure 3). PAME relaxations were not affected by removal of the endothelium (Figure 3C). Pre-treatment of aortic rings with XE991 (30 μM, 30 min) prevented the relaxant effects of PAME (Figures 3B,F). PAME effects were not abolished by the K_v_ channel blocker 4-aminopyridine (4-AP 2 mM, 10 min) (Figure 3D) or NDGA (10 μM, 30 min), a lipoxygenases inhibitor (Figure 3G). The cytochrome P450 epoxygenase inhibitor 17-octadecynoic acid (ODYA 10 μM, 30 min) slightly inhibited PAME relaxations in rat aorta (Figure 3E). However, the soluble epoxide hydrolase inhibitor 12-(3-adamantan-1-ylureido)-dodecanoic acid (AUDA 10 μM, 30 min) did not increase PAME relaxations (Figure 3H), which is expected for involvement of P450 expoygenase mediators. Instead, it inhibited PAME relaxations implicating non-specific effects of ODYA in inhibiting PAME effects. PAME up to 10 μM failed to induce relaxations of (–) fat human mesenteric arteries (Figure 4). Pre-treatment of the vessels with XE991 (30 μM, 30 min) did not affect the lack of PAME effects (Figures 4B,C). Taken together, the results indicate that exogenous PAME at relatively high concentrations (EC_50_ >1 μM) can induce slight (E_max_ ~25%) relaxations in pre-contracted rat aortic rings, which are mediated by opening of K_v_7 channels. In contrast, PAME is not a potent vasodilator (up to 10 μM) of human mesenteric arteries.

### Role of PAME in ADRF-containing bath solutions of rat aortas and contribution of K_v_7 channels

**Figure 5:**
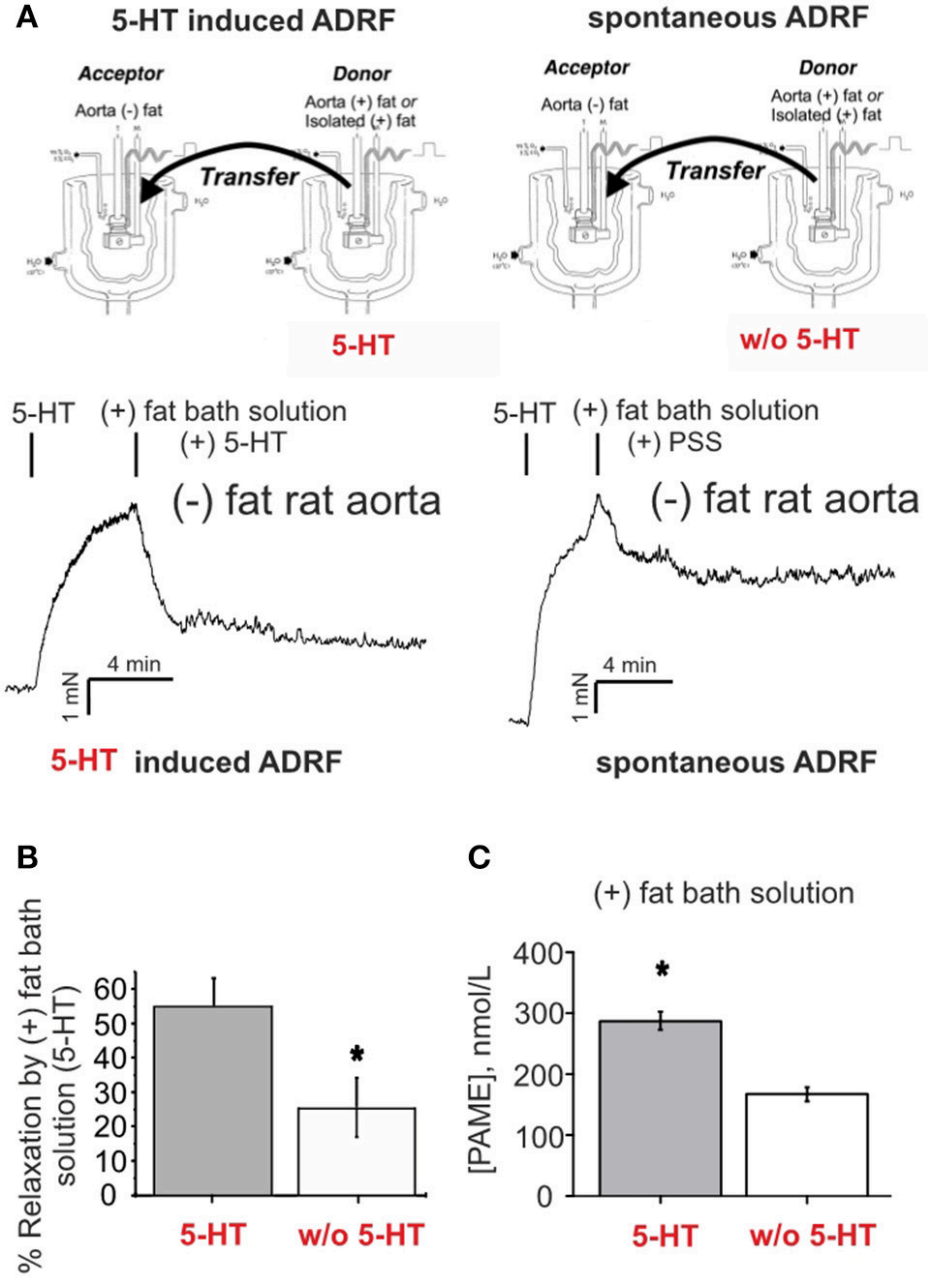
Relaxation of (–) fat aortic rings induced by transfer of bath solutions from (+) fat rat aortic rings. **(A)** Original recordings demonstrating stronger relaxations of ADRF-containing bath solutions from (+) fat rings incubated with 5-HT than from (+) fat rings incubated in PSS without 5-HT. **(B)** Bath solutions from (+) fat rings incubated with 5-HT produced stronger relaxations than bath solutions from (+) fat rings incubated in PSS without 5-HT. Relaxation is expressed as a percentage of the steady-state tension (100%) obtained with 2 μM 5-HT. Data are expressed as mean ± SEM of 6 (left bar) and 5 rings (right bar) prepared from 6 and 5 animals, respectively. **(C)** Bath solutions from rat peri-aortic adipose tissue incubated in the presence 5-HT (*n* = 8) produced more PAME than bath solutions from rat peri-aortic adipose tissue incubated in the absence of 5-HT (*n* = 8). ^*^p < 0.05.

To demonstrate that the intact aortic preparation releases ADRF which can abrogate vascular contraction by opening VSMC K_v_7 channels (Tano et al., 2014; Gollasch, 2017), we performed bioassay experiments where we transferred aliquots of the bath solution from an intact donor preparation incubated in 2 μM 5-HT-containing solution to vessel preparations without PVAT, pre-contracted with 5-HT. This maneuver transferred the factor (Tano et al., 2014; Gollasch, 2017) released by either intact preparations or isolated perivascular adipose tissue (PVAT) (Figure 5B) to arteries without adipose tissue (Tano et al., 2014). Bath solutions from (+) fat rings incubated with 5-HT produced stronger relaxations than bath solutions from (+) fat rings incubated in PSS without 5-HT (Figures 5A right, B), indicating that ADRF release is increased by 5-HT. According to the proposed K_v_7 channel mechanism (Tano et al., 2014; Gollasch, 2017), ADRF produced relaxations, which were inhibited by XE991 (30 μM) (Supplementary Figures 1). Next, we were interested in [PAME] in ADRF-containing bath solutions and whether PAME release from PVAT can be stimulated by 5 μM 5-HT. We found that 5-HT is capable to release endogenous PAME from rat aortic PVAT samples (Figure 5C). However, the PAME concentrations in 10 mL PSS solutions containing 3 g rat aortic PVAT samples were lower than 300 nM, indicating that effective PAME concentrations in myograph bath chambers are in the range of < 1 nM (i.e., 500 times lower). Figures 5C, 3A–H show that [PAME] lower than 1 nM are unable to affect wall tension to produce relaxations of (–) rat aortas. These data indicate that PAME cannot be the transferable ADRF we seek.

### Endogenously released PAME levels in bath solutions of human visceral adipose tissue are low and not controlled by 5-HT

Endogenously released nominal PAME levels were also detected in aliquots of bath-solutions containing 3 g human visceral adipose tissue in 10 mL PSS (Figure 4D). However, in contrast to the rat aorta (Figure 5C), PAME levels were even lower and 5-HT (5 μM) was unable to induce endogenous PAME release in the adipose tissue samples. These data indicate that 5-HT can induce PAME release from rat peri-aortic adipose tissue, but not from human visceral adipose tissue, where spontaneous PAME release is even lower and not controlled by 5-HT.

## Discussion

In the present study, we investigated the roles of K_v_7 channels and PAME in PVAT regulation of arterial tone in human visceral mesenteric arteries. The major findings are that XE991-sensitive K_v_ (K_v_7) channels are involved in the anti-contractile effects of PVAT on human mesenteric arteries, similarly to rat aortas. Furthermore, exogenous PAME displays properties of a PVRF in rat aorta, where it may contribute to paracrine PVAT regulation of arterial tone, but not in human mesenteric arteries. Our data indicate that PAME is not ADRF. Nevertheless, the data support previous findings (Lee et al., 2011) suggesting that fatty acids, particularly perivascular adipose tissue-derived methyl palmitate (PAME), can play a role in paracrine regulation of vascular tone and possibly in the pathogenesis of hypertension in rats, where deficiency or malfunction of K_v_ channels (Gálvez et al., 2006; Galvez-Prieto et al., 2008), particularly K_v_7 channels (Jepps et al., 2011; Li et al., 2013; Zavaritskaya et al., 2013), have been suggested to be involved. Our results suggest that these effects could involve malfunctional K_v_7 channels, independently of metabolism of endogenous lipid epoxides. Since the [PAME] released into bath media were exceptionally low, we conclude that PAME released from PVAT only in close proximity to VSMCs can regulate arterial tone in rat aorta.

### PVAT effects and K_v_7 channels

We demonstrated earlier that PVAT markedly attenuates the contractile response to 5-HT, phenylephrine and angiotensin II in aortic and mesenteric ring preparations of rats (Löhn et al., 2002; Verlohren et al., 2004). The data suggest a major role of the K_v_7 family of K^+^ channels as putative downstream targets of ADRF, which is a major PVRF released from PVAT to reduce arterial tone (Zavaritskaya et al., 2013; Gollasch, 2017). This suggestion is supported by findings showing that XE991 (K_v_7 blocker) inhibited the anti-contractile effects of PVAT in visceral arteries of rats and mice (Schleifenbaum et al., 2010; Tano et al., 2014) (this study). Data were obtained by two different vasoconstrictor agonists, namely 5-HT and phenylephrine, indicating that K_v_7 channel targeting could be common mechanism for PVAT regulation of arterial tone. We employed XE991 at 30 μmol/L to ensure effective block of the K_v_7 channels because native VSMC K_v_7.4 and K_v_7.5 channels are inhibited by this compound with IC_50_ of 5.5 and 65 μmol/L, respectively (Tykocki et al., 2017). The XE991 effects are unlikely mediated by inhibition of BK_Ca_ or K_v_7.1 channels (Tsvetkov et al., 2016b, 2017). Our present results show that PVAT displayed anti-contractile effects in human mesenteric arteries. The anti-contractile effects were inhibited by XE991, supporting the idea that K_v_7 channels are involved in PVAT regulation of arterial tone in humans. Data obtained on *Kcnq1*^−/−^ mice (Tsvetkov et al., 2016b) suggest that these effects are mediated by K_v_7 channels distinct from K_v_7.1, i.e., most likely K_v_7.3, K_v_7.4 and/or K_v_7.5, which are all expressed in mesenteric artery VSMCs from rats (Mackie et al., 2008; Jepps et al., 2011; Zavaritskaya et al., 2013), mice (Yeung et al., 2007; Tsvetkov et al., 2016b, 2017), and humans (Ng et al., 2011).

### PAME effects and K_v_7 channels

We found that PAME was capable to produce relaxations of rat aortas. These effects were inhibited by XE991. The effects were not inhibited 2 mM 4-AP, but see (Lee et al., 2011). In contrast, PAME at similar concentrations did not relax human mesenteric arteries. Together, these data suggest that PAME could contribute to PVAT relaxations by activating K_v_7 channels in rat aorta, but not in human mesenteric arteries. The results are in line with the idea that 4-AP is not inhibiting K_v_7 channels in rat aorta. We next explored the role of PAME and K_v_7 channels in the anti-contractile effects of PVAT in rat aorta using a bioassay approach (Gálvez et al., 2006; Galvez-Prieto et al., 2008). In these experiments, we confirmed earlier findings indicating that 5-HT induces vessel relaxation by releasing a transferable vasoactive substance (ADRF) from PVAT into the bath solution (Maenhaut and Van de Voorde, 2011; Gollasch, 2012). As a negative control, we transferred aliquots of periadventitial fat solution in a similar fashion without 5-HT. We found that these aliquots produced less potent relaxations in rat aortas without PVAT suggesting that 5-HT is capable to stimulate the release of ADRF from PVAT. Moreover, inhibition of K_v_7 channels in (–) fat aortic rings by XE991 disrupted these effects in our bioassay experiment. We previously demonstrated that ADRF effects occur without involvement of the endothelium (Gollasch, 2012). Thus, the present data indicate that 5-HT induces ADRF release from PVAT, which displays anti-contractile properties though activation of XE991-sensitive (K_v_7) K_v_ channels in VSMCs. We next focused on PAME bioactivity and release under these conditions. This part of our study is important for understanding the role of PAME as putative ADRF and/or paracrine PVRF. We found that PAME (EC_50_ ~1.4 μM) only slightly relaxed rat aortas (E_max_, about 25%), whereas similar concentrations of PAME had no effects on human mesenteric arteries.

### PAME source and metabolism

Palmitic acid, or hexadecanoic acid in IUPAC nomenclature, is the most common saturated fatty acid found in plants, animals and humans. Together with stearic acid and oleic acid, palmitate acid belongs to the free fatty acids (FFAs), which play an important role as a source of energy for the body (Ibarguren et al., 2014). Endogenous PAME appears to play a role in modulation of the autonomic ganglionic transmission and vasodilatory effects of nitric oxide (NO) (Lin et al., 2008). In plants, palmitate acid can be metabolized through the lipoxygenase pathway (Osipova et al., 2010). However, there is no evidence that palmitate acid is metabolized through the lipoxygenase pathway in animals or humans. Although palmitate acid seems to have some effects on lipoxygenase and cyclooxygenase in platelets (Sakai et al., 1976), there are no reports that this occurs in other mammalian cells. Consistently, it is not surprising that NDGA, a non-selective lipoxygenase inhibitor, failed to inhibit exogenous PAME relaxation in rat aorta in our study, suggesting no involvement of lipoxygenases metabolites in the PAME effects in the vasculature.

Dietary triacylglycerols with palmitic acid can reduce plasma phospholipid arachidonic and docosahexaenoic acids *in vivo* (Innis and Dyer, 1997). To rule out involvement of cytochrome P450 (CYP) metabolites in PAME relaxation in rat aorta, we tested the effects of a CYP epoxygenases and FFA ω-hydrolases inhibitor (ODYA), and a soluble epoxide hydrolase inhibitor (AUDA), which blocks breakdown and inactivation of CYP–derived active vasodilatory metabolites from arachidonic acid, linoleic acid, eicosapentaenoic acid and docosahexaenoic acid (Hercule et al., 2007, 2009). Since ODYA (10 μM) and AUDA (10 μM) did not produce reciprocal effects on PAME relaxations, we conclude that PAME is a vasodilator in rat aorta independently of metabolism of endogenous lipid epoxides.

### PAME released by PVAT

Most vessels possess some amount and type of PVAT, varying from mostly brown fat (thoracic aorta) to mixed brown and white fat (mesenteric vessels) (Watts et al., 2013). We were able to detect endogenously released PAME in bathing solutions of both rat peri-aortic and human visceral adipose tissues. Furthermore, 5-HT was capable to induce PAME release from rat peri-aortic adipose tissue, indicating a humoral active release process. However, the measured nominal concentrations of PAME were too low to explain transferable ADRF properties in both vascular preparations under study. Together, we suggest that PAME is actively released from PVAT and displays properties of relaxing factor in rat aorta, but not in human mesenteric arteries, where it may contribute as paracrine PVRF to PVAT regulation of arterial tone, independently of metabolism of endogenous lipid epoxides. It will be interesting to determine human mesenteric PVAT is unresponsive to PAME release and action because it is mostly white adipose tissue.

In conclusion, our studies implicate important roles of K_v_7 channels in PVAT control of arterial tone in both rat aorta and human mesenteric arteries, which supports previous findings obtained on other, non-human arteries (Gollasch, 2017). Furthermore, our study highlights the potential role of PAME to contribute as paracrine PVRF to regulation of vascular contraction by opening K_v_7 channels, at least in rats. Our study has translational implications since malfunction of PVAT/K_v_7 channels has been proposed to contribute and to serve as therapeutic targets to improve vascular dysfunction in experimental obesity and hypertension (Rahmouni, 2014), but data on existence of this prototype of vasoregulation in human vessels were missing. Further studies are warranted to investigate PVRFs, K_v_7 and other vascular potassium channels to develop new prevention and treatment strategies for cardiovascular disorders associated with obesity and hypertension.

## Author contributions

All authors planned and designed the experimental studies. NW, AK, and GD performed the wire myography experiments. NW and MG drafted the article, and all authors, contributed to its completion.

### Conflict of interest statement

The authors declare that the research was conducted in the absence of any commercial or financial relationships that could be construed as a potential conflict of interest.

## Supplementary material

Supplementary Figure S1 Original recordings demonstrating relaxation of (–) fat rat aortic rings by transfer of ADRF-containing bath solutions from (+) fat rat aortic rings **(A)**, and pre-treatment of (–) fat vessels with 30 μM XE991 can inhibit relaxation of transfer ADRF-containing bath solutions in (–) fat rat aortic rings **(B)**.

Supplementary Figure S2 Presence of anti-contractile effects of perivascular fat in human mesenteric arteries and their inhibition by XE991. **(A,B)** Original recordings showing inhibition of the anti-contractile effects of perivascular fat by XE991 (30 μM). Recordings are shown in the presence (red curves) and absence (black curves) of XE991. XE991 had no effects on phenylephrine contractions in (–) fat rings **(A)**, but increased phenylephrine contractions in (+) fat rings **(B)**. **(C)** Concentration-response curves for phenylephrine in (+) fat vessels in the presence (*n* = 23) and absence of XE991 (*n* = 18) and in (–) fat vessels in the presence (closed circle, *n* = 25) and absence of XE991 (*n* = 17). ^*^*p* < 0.05 for (+) fat control vessels compared to all other groups each.
