# Supplementary figures and images for: Palmitic Acid Methyl Ester and Its Relation to Control of Tone of Human Visceral Arteries and Rat Aortas by Perivascular Adipose Tissue
Source: Front Physiol. 2018 May 23;9:583. doi: 10.3389/fphys.2018.00583 (PMC5974537; doi:10.3389/fphys.2018.00583)

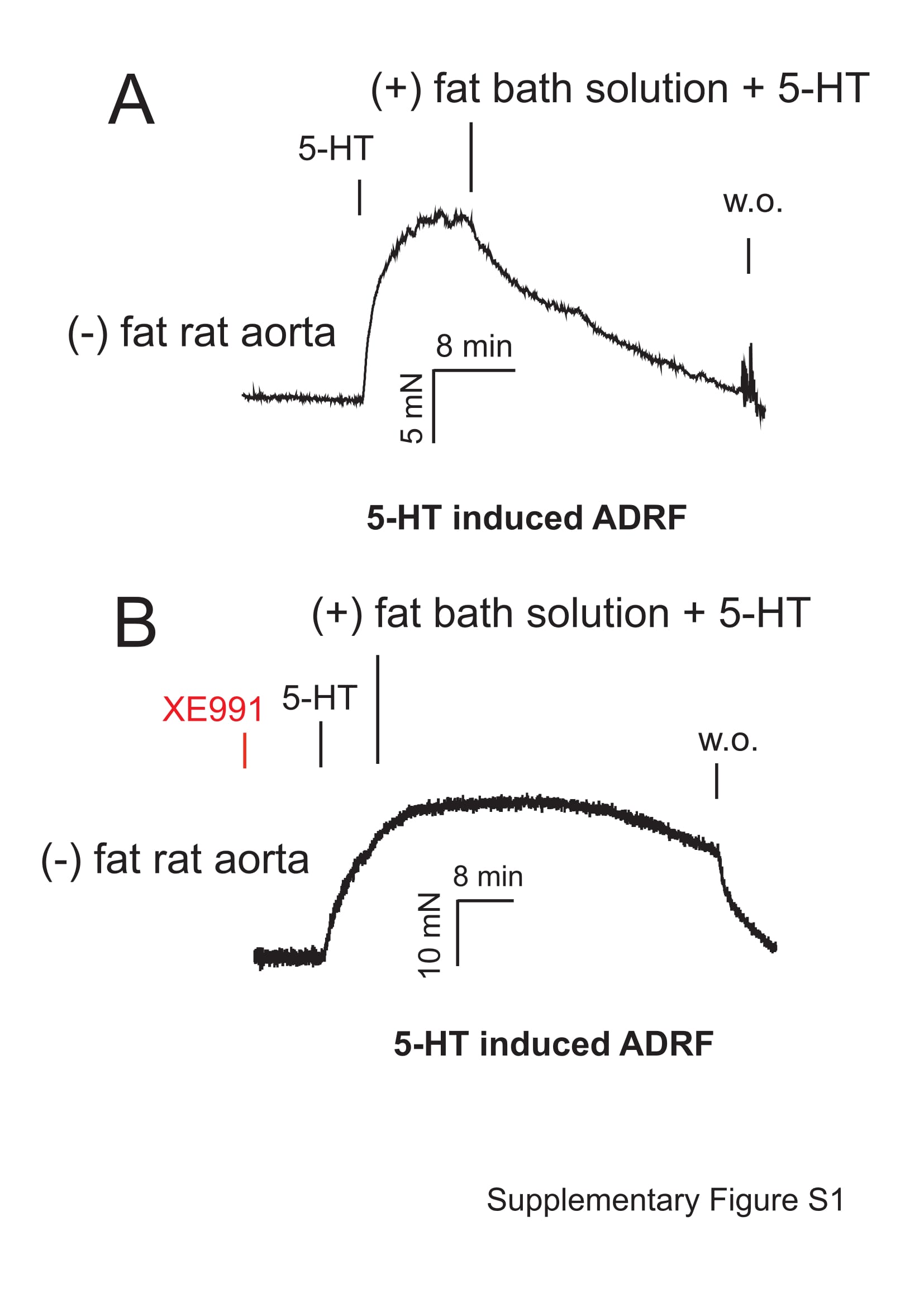

Supplement: Supplementary Figure S1 — Original recordings demonstrating relaxation of (–) fat rat aortic rings by transfer of ADRF-containing bath solutions from (+) fat rat aortic rings (A), and pre-treatment of (–) fat vessels with 30 μM XE991 can inhibit relaxation of transfer ADRF-containing bath solutions in (–) fat rat aortic rings (B). [file Image_1.jpeg]

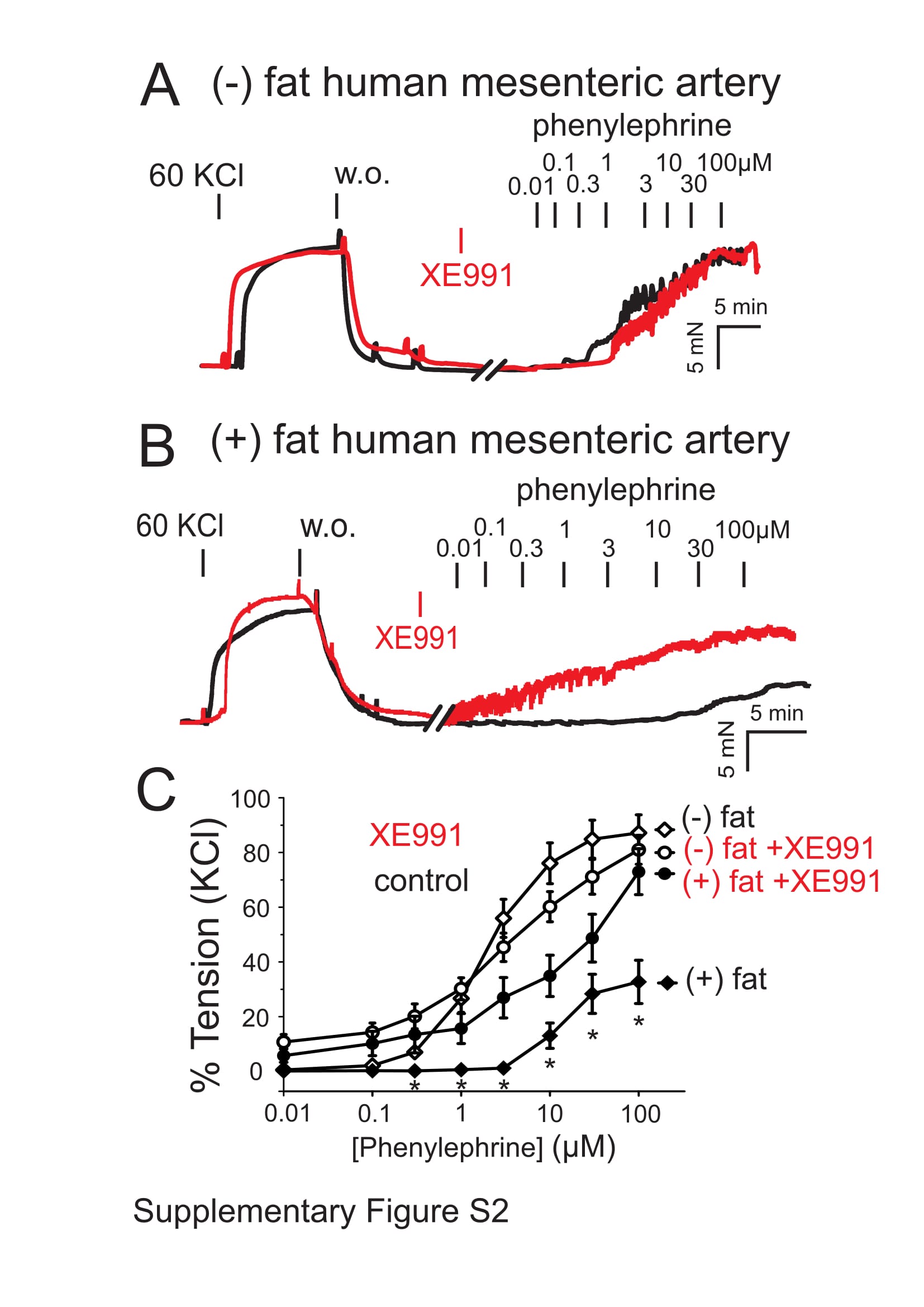

Supplement: Supplementary Figure S2 — Presence of anti-contractile effects of perivascular fat in human mesenteric arteries and their inhibition by XE991. (A,B) Original recordings showing inhibition of the anti-contractile effects of perivascular fat by XE991 (30 μM). Recordings are shown in the presence (red curves) and absence (black curves) of XE991. XE991 had no effects on phenylephrine contractions in (–) fat rings (A), but increased phenylephrine contractions in (+) fat rings (B). (C) Concentration-response curves for phenylephrine in (+) fat vessels in the presence (n = 23) and absence of XE991 (n = 18) and in (–) fat vessels in the presence (closed circle, n = 25) and absence of XE991 (n = 17). *p < 0.05 for (+) fat control vessels compared to all other groups each. [file Image_2.jpeg]
